# Brief Description of the Shedding of Temporary Teeth, as to Time, Order and Process

**Published:** 1889-01

**Authors:** H. H. Schuman

**Affiliations:** U. of M.


### A Brief Description of the Shedding of Temporary Teeth, as to Time, Order and Process.

BY H. H. SCHUMAN, U. OF M.

I will ask your attention and consideration to the following remarks upon "A brief description of the shedding of the temporary teeth in regard to time and order."

When the small size and delicate structure of the jaws of an infant, and the fact that the teeth correspond to them in size, are considered, it will be apparent that the provision of a second set, large and strong in proportion to the increased size and strength of the adult jaw, is a necessity.

Almost coincidently with the development of the jaw for the temporary tooth appears the germ for its permanent successor. As these (the permanent teeth) approach their full development, a process called "absorption" is set up, by which the roots of the temporary teeth are little by little dissolved off and the particles constituting them are carried off, and nothing but the crown remains. This process (which will be considered later more fully) does not begin in all the teeth at the same time, but in the order corresponding to their development and eruption.

There can be no definite rule laid down as to the exact time of the shedding of each temporary tooth, as there are many reasons for variation in time and order of their being replaced by permanent successors.

External as well as internal changes in the tooth itself and surrounding structure are direct causes for their being shed. It quite frequently happens that one, two or all of the temporary teeth are retained weeks and even *months* too long, or shed weeks or months too soon. By considering one of the causes of the shedding of the temporary teeth briefly,(as I will take this subject up more minutely in my next paper) we can easily see how a tooth may be lost before its time.

For instance decay. It attacks the teeth beyond its origin and even attacks the periosteum. You all have already seen teeth elongated or become loose from mere decay, which not even extended to the pulp. If such decay then attacks the periosteum, to a sufficient degree, long before the regular shedding time of the tooth, such a tooth would be lost long before its time. Dr. White says quite right :

" Parents are undoubtedly greatly to be blamed for the indifference they sometimes show for the care of the temporary teeth of their children. Unfortunately the dentist is rarely consulted until pain in some of these organs brings the little sufferer to him for relief."

The result then quite frequently is the loss of the tooth, and one of the bad effects following it and irregularity of the permanent set. I will not go into this subject further, as it will be more fully discussed in the paper on the process of shedding of temporary teeth.

It sometimes happens that temporary teeth maintain their place to the exclusion of permanent ones, which are then kept within the substance of the jaw, or appear in some irregular position.

The lower incisors are the first to become loose and fall out, then the upper ones follow, then the laterals ; the lower ones as a general rule preceding the upper ones, and so on in order in which they appeared, so that that tooth which has made its appearance first goes first, and which comes last goes last.

Let us compare the time of eruption of the temporary teeth with their respective time of shedding, and see what conclusions we can draw from such a comparison.

The central incisors come between 8 and 9 months and go at 6 and 8 years. Lateral incisors come between 9 and 10 months and go at 7 and 9 years. Canines come between 12 and 16

months and go at 11 and 13 years. Molars come between 14 and 20 months and go between 8 and 12 years.

We have then the central incisors lasting on an average 66 months. The lateral incisors 75 months. The molars 90 months. cuspids 120 months.

The cuspids as you see lasting longer generally, than any of the others. The question naturally arises, why this tooth should last longer than any of the others. I shall try to explain my reasons for this as well as I can: In my own mind the reason seems pretty clear, but I do not know whether I will be able to convey my ideas as well as I would like to. (I find in the American System of Dentistry). "In the following developments of the deciduous teeth from the appearance of the epithelial eminence, the epithelial inflection, and the epithelial band or enamel organ, we recognize various progressive changes in the germinal area between the 7th and 17th week, the modifications during this period in these structures are for the development of 20 temporary teeth. The further development of these 20 germs after the 17th week of fœtal life simply serves to give each gradually its respective form and hardness. Now the incisors are fully formed (but yet in a soft condition) at about the 40th week of fœtal life, from then until the 8th or 9th month after birth, gradually the roots are formed and the calcium salts are deposited in them, giving them their hardness.

Now let us compare these with the cuspids and molars.

Already at the 6th month of fœtal life they are fully formed, and from then until the 16th and 20th month the calcium salts are being deposited in them. That is, the incisors have but 20 and 30 weeks time for the process of hardening (that for the deposition of lime salts). The cuspids and molars have 74 weeks, and during that time receive a far greater density than the incisors. Therefore they are far better protected against any external influence, and any internal destructive agents will not be able to destroy them as easily and quickly as they would the incisors. Another reason why the cuspids are protected so much more than the incisors from any external destructive agent, is their crowns being conical, very convex externally, and therefore easily self-cleansing.

Now let us examine a temporary tooth, which has been thrown off. There are no roots left and the pulp chamber, if any left, is generally found open, and is not like that of a perfect organ completely enveloped, but has for a greater or less time communicated freely, and directly with the surrounding soft parts.

A knowledge of this is very valuable, as it will guard us against a treatment of aching deciduous teeth at certain times, which at others may be very beneficial, but during this period it would do but little good, and it may be harm. Well, if we find the pulp chamber open, we naturally would ask what has become of the pulp.

I will leave this subject here and let you gentlemen consider it a little, and will endeavor to answer this question in a brief description of the process of "shedding of temporary teeth" in my next paper.

